# Using additional pressure control lines when connecting a continuous renal replacement therapy device to an extracorporeal membrane oxygenation circuit

**DOI:** 10.1186/s12882-018-1172-2

**Published:** 2018-12-19

**Authors:** Soo Jin Na, Hee Jung Choi, Chi Ryang Chung, Yang Hyun Cho, Hye Ryoun Jang, Gee Young Suh, Kyeongman Jeon

**Affiliations:** 1 Department of Critical Care Medicine, Samsung Medical Center, Sungkyunkwan University School of Medicine, Seoul, Republic of Korea; 2 Intensive Care Unit Nursing Department, Samsung Medical Center, Sungkyunkwan University School of Medicine, Seoul, Republic of Korea; 3 Department of Thoracic and Cardiovascular Surgery, Samsung Medical Center, Sungkyunkwan University School of Medicine, Seoul, Republic of Korea; 4 Division of Nephrology, Department of Medicine, Samsung Medical Center, Sungkyunkwan University School of Medicine, Seoul, Republic of Korea; 5 Division of Pulmonary and Critical Care Medicine, Department of Medicine, Samsung Medical Center, Sungkyunkwan University School of Medicine, 81 Irwon-ro, Gangnam-gu, Seoul, 06351 Republic of Korea

**Keywords:** Renal replacement therapy, Extracorporeal membrane oxygenation, Critical care, Acute kidney injury

## Abstract

**Background:**

The introduction of a continuous renal replacement therapy (CRRT) device into the extracorporeal membrane oxygenation (ECMO) circuit is widely used. However, excessive pressure transmitted to the CRRT device is a major disadvantage. We investigated the effects of using additional pressure control lines on the pressure and the lifespan of the CRRT circuit connected to the ECMO.

**Methods:**

This is an observational study using prospectively collected data from consecutive patients receiving CRRT connected into the ECMO circuit at a university-affiliated, tertiary hospital from January 2013 to December 2016. The CRRT circuit was connected into the ECMO circuit through the Luer Lock connection without an additional pressure control line in 16 patients (9%, no line group), an additional pressure control line on the inlet line in 36 patients (23%, single line group), and additional pressure control lines on both the inlet and outlet lines in 118 patients (77%, double line group). The outcome measures of interest were compared among the three groups.

**Results:**

The median access pressure was higher in the no line group compared to the groups. However, median filter pressure, effluent pressure, and return pressure were higher in the double line group compared to the other groups. There were no significant differences in platelets, lactate dehydrogenase, and plasma hemoglobin among the 3 groups over the time period studied. Median lifespan of the CRRT circuits in the double line group was 45.0 (29.0–63.7) hours, which was higher compared to 21.8 (11.6–31.8) hours in the no line group and 23.0 (15.0–34.6) hours in the single line group, respectively. In addition, in-hospital mortality was lower in the double line group (48.3%) compared to the no line group (68.8%) and the single line group (75.0%).

**Conclusions:**

Additional tubing can be considered a simple and safe method for pressure control and lengthening circuit survival when connecting the CRRT device to the ECMO circuit.

## Background

Acute kidney injury (AKI) and fluid overload frequently develop in critically ill patients receiving extracorporeal membrane oxygenation (ECMO) support for severe cardiopulmonary insufficiency [1, 2] and are associated with poor prognosis [3, 4]. Continuous renal replacement therapy (CRRT) provides an efficient and potentially beneficial method of renal replacement and fluid management in patients receiving ECMO support [5, 6], however, combining these two separate extracorporeal circuits remains a challenge [5–7]. Conceptually, the idea of connecting the CRRT device to the ECMO circuit is a convenient way to operate the CRRT device without additional catheter insertion, but in practice, there are several technical concerns. One of the major disadvantages of incorporating the CRRT device into the ECMO circuit is excessive positive or negative pressure transmitted to the CRRT device, resulting in unpredictable consequences [8]. Although several studies suggested measures to handle this problem [8–11], no standardized method exists.

Pressure is the product of flow and resistance, and both are influenced by various factors, such as diameter and length of the conduit and fluid viscosity [12]. Therefore, introducing additional tubing between the CRRT device and the ECMO circuit when connecting these two extracorporeal systems is a simple method to reduce blood flow between the systems and, ultimately, control the excessive pressures [11]. However, there is limited information available on the effects of this method on CRRT in the clinical setting. The objective of this study was to investigate the effects of using additional pressure control lines on the pressure and the lifespan of the CRRT circuit in adult patients receiving CRRT connected to the ECMO circuit.

## Methods

### Study population

**Fig. 1:**
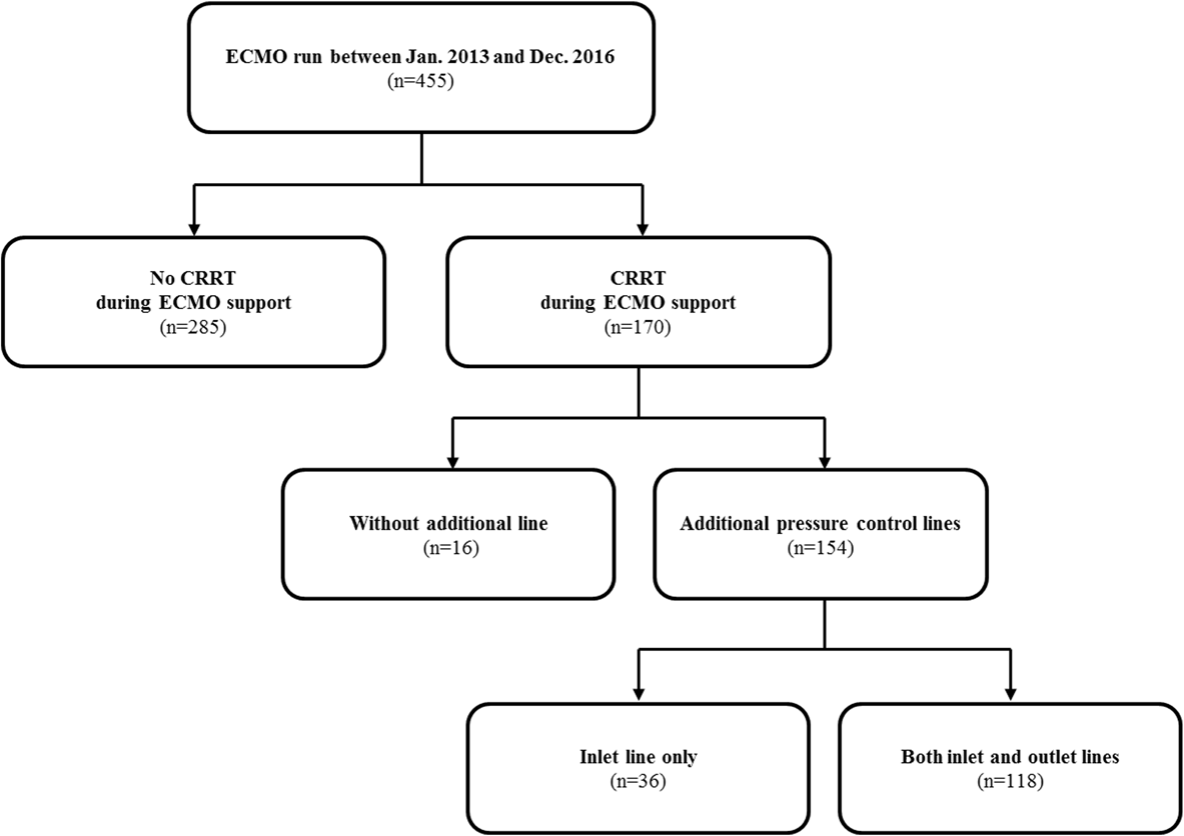
Scheme of group distribution

We reviewed consecutive adult patients treated with ECMO for circulatory or respiratory failure at Samsung Medical Center in Seoul, South Korea between January 2013 and December 2016. A total of 455 ECMO runs in 431 patients were identified during the study period. After excluding 261 patients who did not receive CRRT during ECMO support, we investigated 170 patients in whom the CRRT device was connected into the ECMO circuit (Fig. 1). The institutional review board of Samsung Medical Center approved this study and waived the requirement for informed consent because of the observational nature of the study.

### ECMO equipment

Patients with circulatory or respiratory failure refractory to conventional therapy were considered as candidates for ECMO [3]. Mode and cannulation were determined according to the patient’s condition. A 20–28-Fr multi-stage venous cannula was used for drainage via the common femoral vein, and a 14–18-Fr or 20–24-Fr short cannula was used for venous return via the internal jugular or the common femoral vein, respectively. In venoarterial mode, a 14–24-Fr cannula was inserted into the femoral artery. The Prolonged Life Support System (Quadrox PLS, Maquet Inc., Rastatt, Germany) and the Capiox Emergency Bypass System (Capiox EBS; Terumo, Inc., Tokyo, Japan), which includes a centrifugal pump and heparin-coated polypropylene hollow fiber membrane oxygenator, were available in our hospital. Pump blood flow and sweep gas flow rates were adjusted to maintain optimal tissue perfusion and gas exchange. Anticoagulation was achieved by intravenous heparin titrated to an activated clotting time between 180 and 220 s or activated partial thromboplastin time between 55 and 75 s. Argatroban was used as an alternative anticoagulant when heparin-induced thrombocytopenia was suspected or confirmed. Anticoagulant was stopped in the presence of active bleeding at the discretion of the attending physician. Fluids or drugs were administered directly to the patient’s venous line, and the ECMO circuit was not used for this purpose. Pre-pump venous drainage, pre-membrane pressures, and post-membrane pressures were not routinely measured.

### Connection of CRRT lines to ECMO circuit

**Fig. 2:**
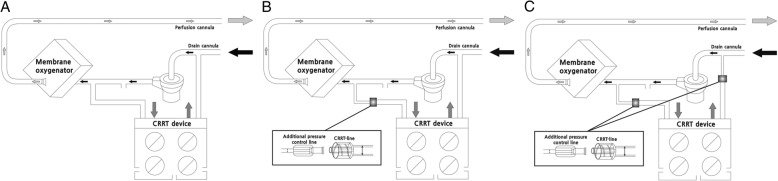
Schematic of the connection of the continuous renal replacement therapy (CRRT) device and the extracorporeal membrane oxygenation (ECMO) circuit. **a**. The CRRT circuit is connected into the ECMO circuit through the Luer Lock connection without additional pressure control lines, The two circuits are directly connected by configuring the inlet (access) line of the CRRT device to the post-pump ECMO circuit and the outlet (return) line of the CRRT device to the pre-pump ECMO circuit. **b**. An additional pressure control line is added on the inlet line of the CRRT circuit. **c**. Additional pressure control lines are added on both the inlet and the outlet lines of the CRRT circuit

Before 2013, our institution performed CRRT through a separate vascular access independent of the ECMO circuit. Since 2013, however, we have directly connected the CRRT device into the ECMO circuit in the following manner: inlet (access) line of the CRRT device to post-pump ECMO circuit and outlet (return) line of the CRRT device to the pre-pump ECMO circuit through the Luer Lock connection [8]. Prior to July 2013, we did not utilize an additional line in the connection of the CRRT device and the ECMO circuit (Fig. 2a). In July of 2013, we connected the inlet line of the CRRT circuit to the port of the post-pump ECMO circuit with an additional pressure control line (inner diameter 1.5 mm, length 30 cm; Hyupsung Medical Co., Gyeonggi-do, South Korea) to manage excessive pressure on the inlet line of the CRRT device transmitted from the positive pressure part of the ECMO circuit. The outlet line was connected to the port of the ECMO circuit at the pre-pump ECMO circuit without an additional pressure control line (Fig. 2b). After November 2013, we modified the connection of the outlet line to the port of the ECMO circuit at the pre-centrifugal pump, adding an additional pressure control line similar to the connection of the inlet line of the CRRT circuit to the ECMO circuit (Fig. 2c).

### Management of CRRT

CRRT was performed using the Prismaflex system (Baxter International, Deerfield, IL, USA) and the Prismaflex ST100 circuit. All Prismaflex systems installed with software, FLEX version 8.1. The priming volume was 152 mL. All patients were dialyzed in CVVHDF (continuous veno-venous hemodiafiltration) modality. Commercially prepared bicarbonate-buffered replacement fluid was used as dialysate and replacement fluid. The ultrafiltration rate and dialysate flow rate were adjusted according to clinical requirements in the range of 1000–2000 mL/h (25–35 mL/kg/h). Replacement fluid was delivered in predilution mode. Standard anticoagulation in our hospital is intravenous heparinization. If a patient had already received systemic anticoagulation with intravenous heparin or agatroban, anticoagulation was not performed for CRRT. If a patient could not maintain systemic anticoagulation due to bleeding, regional anticoagulation with nafamostat (Futhan®, SK chemicals, Seoul, Korea) for CRRT [13] or discontinuation of anticoagulant was considered. Filter was changed after 72 h of use following manufacturer’s recommendations when CRRT is needed for more than 72 h, or as needed due to filter clotting.

Under supervision of the nephrologist, nurse practitioners and registered nurses were trained to manage the CRRT system and the clinical needs of the patients on CRRT. Bedside nurses were responsible for running, maintaining, and troubleshooting the CRRT system. Orders for CRRT were the responsibility of the nephrologist. The intensive care unit (ICU) physicians were permitted to write orders to adjust fluid removal rates in collaboration with the nephrologist.

### Data collection and clinical outcomes

The following data were collected prospectively from 2013 onward as part of the clinical care for all patients on CRRT in our ICU: patient demographics, comorbidities, acute physiology and chronic health evaluation (APACHE) II score on ICU admission, clinical reason for CRRT initiation, setting and parameters of CRRT, and anticoagulation method. The data were recorded on the first day of CRRT initiation. The inlet and outlet pressures of the CRRT circuit displayed on the CRRT device were recorded prospectively. We used pressure values after initiating CRRT for the first time or changing to a new filter to minimize the influence of the filter condition as much as possible in assessing the effect of ECMO on CRRT depending on the connection method. To address the primary research question of whether additional pressure control lines influence CRRT circuit pressures, we measured the pressure on the added lines as well. Laboratory data were obtained before CRRT initiation, 24 h after CRRT initiation, and 48 h after CRRT initiation. Additional patient data, including illness severity, reasons for ECMO, mode of ECMO, cannulations, and anticoagulation, were extracted from the ECMO registry. ECMO circuit pressures were not monitored during the study period.

The primary outcome in this study was the lifespan of the CRRT circuit. Secondary outcomes were pressures in the CRRT circuit and changes in laboratory findings indicating hemolysis and thrombus formation before and 24 h after CRRT initiation. Clinical outcomes such as rate of weaning from ECMO, duration of ECMO support, adverse events during ECMO, and in-hospital mortality were also identified through medical record review.

### Statistical analysis

Data are presented as median and interquartile range (IQR) for continuous variables and as numbers (percentages) for categorical variables. The baseline characteristics and outcome measures of interest were then compared among the three study groups: no additional pressure control line, single line group, and double line group. Data were compared using the Kruskal–Wallis test for continuous variables and the chi-square or Fisher’s exact test for categorical variables. Multiple comparisons were performed to compare each group using Wilcoxon rank sum tests, and Bonferroni correction was used to determine whether multiple comparisons were significant. To adjust for potential confounding factors in the association between additional pressure control line and lifespan of the CRRT circuit, Cox’s proportional hazards analysis was used. Data are presented as adjusted hazard ratios (HRs) with 95% confidence intervals (CI). Kaplan–Meier estimation was used to determine the lifespan curves of the CRRT circuits by different methods, which were then compared using the log-rank test. For all analyses, a two-tailed test with a *P*-value < 0.05 was considered statistically significant. Statistical analyses were performed using STATA version 14.0 (Stata Corp, College Station, TX, USA).

## Results

### Baseline characteristics

The baseline characteristics of the 170 patients who received CRRT during ECMO support are shown in Table 1. Median age was 56 (45–65) years and 103 (67%) patients were male. The median APACHE II on ICU admission was 23 (15–30). Cardiogenic shock (34%) and cardiopulmonary arrest (34%) were the most common reason for ECMO support, followed by respiratory failure (23%) and septic shock (7%). The majority mode of cannulation for ECMO was venoarterial (75%). The median size of drainage and return cannulas were 22 (21–24) Fr and 16 (15–17) Fr, respectively.

**Table 1:** Patient characteristics upon initiation of extracorporeal membrane oxygenation

Characteristics	No. of patients (%) or median (IQR)
Age, years	57 (47–65)
Male	115 (67.6)
Body mass index, kg/m^2^	24.7 (22.3–27.6)
Comorbidities	
Cardiovascular disease	33 (19.4)
Chronic renal failure	22 (12.9)
Asthma/COPD	7 (4.1)
Liver cirrhosis	8 (4.7)
Malignancy	36 (21.2)
Severity of illness on ICU admission	
APACHE II	23 (15–30)
Reasons for ECMO support	
Cardiogenic shock	57 (33.5)
Cardiopulmonary arrest	57 (33.5)
Respiratory failure	37 (21.8)
Septic shock	11 (6.5)
Weaning failure of CPB	4 (2.4)
Hypovolemic shock	3 (1.8)
Other	1 (0.6)
Cannulation for ECMO	
Venoarterial	127 (74.7)
Venovenous	40 (23.5)
Mixed	3 (1.8)
Cannula size	
Drainage cannula, Fr	22 (21–24)
Return cannula, Fr	16 (15–17)
ECMO flow, L/min	3.7 (3.1–4.2)
Anticoagulation	
Unfractionated heparin	107 (62.9)
Argatroban	4 (2.4)
None	61 (35.7)
Laboratory findings on ECMO initiation	
Blood urea nitrogen, mg/dL	34.5 (23.2–55.7)
Creatinine, mg/dL	1.83 (1.26–2.79)
Bicarbonate, mmol/L	17.6 (13.0–21.8)
Potassium, mmol/L	4.2 (3.8–4.9)
Platelets, 10^3^/uL	89 (53–142)
Lactate dehydrogenase (*n* = 152), IU/L	1551 (903–2853)
Plasma hemoglobin (*n* = 50), mg/dL	18.5 (13.0–32.0)

*APACHE II* acute physiology and chronic health evaluation II, *COPD* chronic obstructive pulmonary disease, *CPB* cardiopulmonary bypass, *ECMO* extracorporeal membrane oxygenation, *ICU* intensive care unit, *IQR* interquartile range

### Initiation of CRRT

During the study period, CRRT was initiated in a median of 1 (0–2) day after ECMO initiation. The CRRT circuit was connected to the ECMO circuit through the Luer Lock connection without additional pressure control lines in 16 (9%) patients (no line group). An additional pressure control line was connected on the inlet line of the CRRT circuit in 36 (23%) patients (single line group) and on both the inlet and outlet lines in 118 (77%) patients (double line group).

Comparisons of patient characteristics, CRRT parameters, and laboratory findings at the time of CRRT initiation among the three groups are presented in Table 2. The no line group had a higher incidence of metabolic acidosis as a reason for CRRT initiation compared to the double line group. In addition, the no line group was prescribed higher blood flow, dialysate flow and replacement fluid flow of CRRT compared to the double line group. However, there was no difference in prescribed CRRT doses and fluid balances among the three groups. Regional nafamostat was more commonly used for anticoagulation in the no line group compared to the single line group and the double line group, respectively. However, the majority of patients received systemic heparin for anticoagulation, which was not significantly different among three groups. There was no significant difference in laboratory findings at the time of CRRT initiation, except for serum HCO_3_, which was lower in the single line group compared to the double line group.

**Table 2:** Comparisons of patient characteristics, prescriptions of CRRT, and laboratory findings among the study groups at the time of CRRT initiation

Characteristics	No line group (*n* = 16)	Single line group (*n* = 36)	Double line group (*n* = 118)	*P* value
Age, years	59 (48–65)	54 (36–62)	58 (48–65)	0.241
Male	12 (75.0)	24 (66.7)	79 (66.9)	0.804
Body mass index, kg/m^2^	23 (21–26)	24.5 (22.5–27.4)	25.0 (22.1–28.0)	0.291
ECMO flow, L/min	3.5 (2.6–4.1)	3.5 (3.0–4.2)	3.8 (3.2–4.3)	0.192
Indication for CRRT				
Acute kidney injury	2 (12.5)	8 (22.2)	31 (26.3)	0.461
Volume overload	6 (37.5)	15 (41.7)	59 (50.0)	0.492
Metabolic acidosis	11 (68.8)	20 (55.6)	35 (29.7)	0.001^†‡^
Prescription of initial CRRT				
Blood flow, mL/min	150 (150–150)	150 (150–150)	150 (150–150)	0.013^†^
CRRT dose, mL/kg/hr	40 (31–46)	36 (27–44)	32 (27–44)	0.062
Dialysate flow, mL/hr	1250 (1000–1500)	1000 (1000–1500)	1000 (1000–1500)	0.033
Replacement fluid flow, mL/hr	1250 (1000–1500)	1000 (1000–1500)	1000 (1000–1500)	0.041
Prescribed fluid balance, mL/hr	0 (− 40–0)	0 (−40–0)	−20 (− 43–0)	0.189
Anticoagulation for CRRT				
Systemic heparin	9 (56.3)	22 (61.1)	76 (64.4)	0.792
Regional heparin	0 (0.0)	0 (0.0)	0 (0.0)	–
Regional nafamostat	5 (31.3)	4 (11.1)	3 (2.5)	< 0.001^†^
Systemic argatroban	0 (0.0)	0 (0.0)	4 (3.4)	0.406
None	4 (25.0)	12 (33.3)	37 (31.4)	0.833
Laboratory findings on CRRT initiation				
Blood urea nitrogen, mg/dL	28.2 (18.3–41.8)	29.7 (18.7–55.9)	38.9 (24.2–59.3)	0.105
Creatinine, mg/dL	1.76 (1.42–2.27)	1.78 (1.23–2.95)	1.93 (1.26–3.07)	0.587
Bicarbonate, mmol/L	18.1 (14.2–19.6)	14.7 (11–18.1)	18.9 (13.8–22)	0.034^‡^
potassium, mmol/L	4.0 (3.5–5.2)	4.1 (3.9–4.8)	4.3 (3.8–4.9)	0.675
Platelets, 10^3^/uL	88 (68–128)	102 (49–152)	87 (53–140)	0.945
Lactate dehydrogenase (n = 152), IU/L	2256 (1531–4350)	1451 (799–4442)	1498 (945–2512)	0.276
Plasma hemoglobin (n = 50), mg/dL	48.9 (42.7 – NA)	13.6 (9.8–22.6)	18.5 (13.3–31.3)	0.090

*CRRT* continuous renal replacement therapy, *CVVH* continuous venovenous hemofiltration, *CVVHDF* continuous venovenous hemodiafiltration

Superscripts †, ‡ indicate significant differences (*p* < 0.05) between the no line group and the double line group, and the single line group and the double line group, respectively

### Parameters of CRRT and clinical outcomes according to connection method

The pressure values of the CRRT circuits are presented in Table 3. The median access pressure was higher in the no line group (163 mmHg) compared to the single ling group (− 37 mmHg) and the double line group (2 mmHg). However, median filter pressure, effluent pressure, and return pressure were higher in the double line group compared to the no line group and the single line group. There were no significant differences in the laboratory values of platelets, lactate dehydrogenase, and plasma hemoglobin from CRRT initiation to 24 and 48 h after treatment initiation among the 3 groups (Table 3).

**Table 3:** Comparisons of parameters of CRRT among the study groups after connecting additional pressure control lines

CRRT parameters	No line group (*n* = 16)	Single line group (*n* = 36)	Double line group (*n* = 118)	*P* value
Pressures on CRRT circuit				
Access pressure, mmHg	163 (117–185)	−37 (−65–57)	2 (−42–52)	< 0.001^*†^
Filter pressure, mmHg	−17(−67–24)	−25 (−49–25)	119 (73–167)	< 0.001^†‡^
Effluent pressure, mmHg	−101 (−138 – −24)	−104 (−139 – −59)	19 (−17–60)	< 0.001^†‡^
Return pressure, mmHg	−81 (−108 – −41)	−71 (−108 – −44)	68 (29–107)	< 0.001^†‡^
Transmembrane pressure, mmHg	57 (36–93)	53 (25–78)	58 (46–69)	0.746
Pressure drop, mmHg	36 (28–42)	32 (20–42)	25 (16–33)	0.003^†^
Change in laboratory findings				
∆PLT between H0 and H24, mg/dL	−27 (−68 – −3)	−1 (−51–27)	−15 (−41–7)	0.289
∆PLT between H0 and H48, mg/dL	−41 (−101–4)	4 (−76–13)	−16 (−45–12)	0.427
∆LD between H0 and H24, IU/L	482 (−650–3405)	384 (−109–1580)	−3 (−237–248)	0.069
∆LD between H0 and H48, IU/L	482 (−53–2648)	−22 (−2237–1237)	−20 (−496–263)	0.354
∆pHb between H0 and H24 (*n* = 17), mg/dL	NA	23.5 (9 – NA)	−5 (−20–2)	0.037
∆pHb between H0 and H48 (*n* = 23), mg/dL	NA	12.5 (12 – NA)	3 (−5–8)	0.100
Lifespan of CRRT circuit, hours	21.8 (11.6–31.8)	23.0 (15.0–34.6)	45.0 (29.0–63.7)	< 0.001^†‡^

*CRRT* continuous renal replacement therapy, *LD* lactate dehydrogenase, *pHb* plasma hemoglobin, *PLT* platelets

Superscripts *, †, ‡ indicate significant differences (*p* < 0.05) between the no line group and the single line group, the no line group and the double line group, and the single line group and the double line group, respectively

**Fig. 3:**
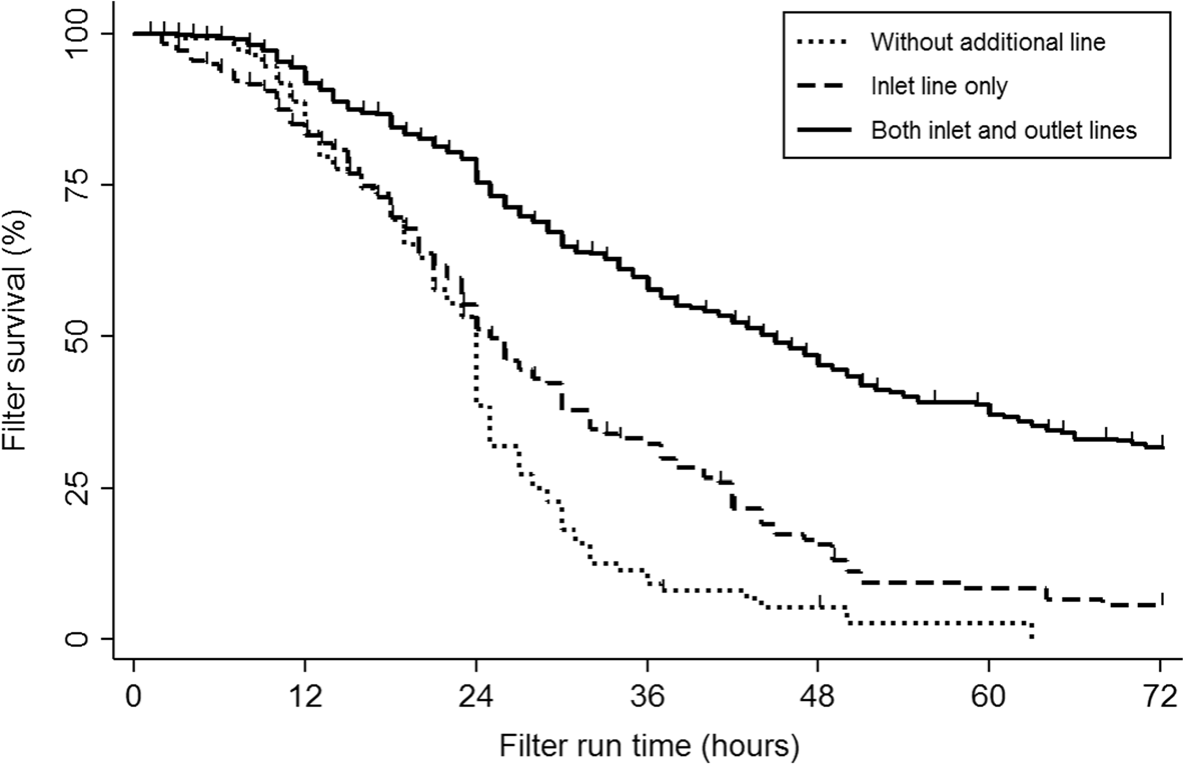
Kaplan–Meier estimate of the lifespan of the continuous renal replacement therapy circuit

The median lifespan of the CRRT circuits in the double line group was 45.0 (29.0–63.7) hours, which was higher compared to 21.8 (11.6–31.8) hours in the no line group (*P* < 0.001) and 23.0 (15.0–34.6) hours in the single line group (*P* < 0.001), respectively. When filter survival rates were compared 72 h after CRRT initiation, the Kaplan-Meier survival estimates showed a significantly higher filter survival rate in the double line group compared to the no line group and the single line group (log rank test, *P* < 0.001) (Fig. 3).

Clinical outcomes of patients who received CRRT during ECMO support are shown in Table 4. There was no difference in ECMO related complications among the three groups. Although the median duration of ECMO support was longer, however, in-hospital mortality was lower in the double line group compared to the single line group.

**Table 4:** Comparisons of clinical outcomes among the study groups after connecting additional pressure control lines

Clinical outcomes	No line group (*n* = 16)	Single line group (*n* = 36)	Double line group (*n* = 118)	*P* value
Duration of ECMO support, hours	80 (53–467)	93 (32–189)	184 (93–501)	0.003^‡^
Successful weaning from ECMO	6 (37.5)	17 (47.2)	72 (61.0)	0.103
ECMO complication				
Gastrointestinal bleeding	2 (12.5)	1 (2.9)	16 (13.8)	0.201
ECMO site bleeding	2 (12.5)	8 (22.9)	15 (12.9)	0.339
Cerebral infarction	2 (12.5)	4 (11.4)	3 (2.6)	0.053
Sepsis	3 (18.8)	5 (14.3)	10 (8.6)	0.356
In-hospital mortality	11 (68.8)	27 (75.0)	57 (48.3)	0.010^†‡^

*ECMO* extracorporeal membrane oxygenation

Superscripts †, ‡ indicate significant differences (*p* < 0.05) between the no line group and the double line group, and the single line group and the double line group, respectively

The results of univariable and multivariable analyses with the proportional hazards regression model for probability of circuit change within 72 h are presented in Table 5. After adjusting for potential confounding factors, the double line group was still significantly associated with lower change of the CRRT circuit (adjusted HR 0.39, 95% CI 0.25–0.60). Other factors independently associated with circuit change within 72 h were type of ECMO, anticoagulation, filter pressure, and return pressure in the CRRT circuits.

**Table 5:** Univariable and multivariable analyses with Cox’s proportional hazards model for probability of circuit change within 72 h

	Univariable		Multivariable			
	HR	95% CI	*P* value	aHR	95% CI	*P* value
Additional pressure control line						
No line	–	–	–	–	–	–
Single line	0.69	0.53–0.91	0.008	0.74	0.46–1.19	0.216
Double line	0.29	0.23–0.37	< 0.001	0.39	0.25–0.60	< 0.001
ECMO type						
Venoarterial	–	–	–	–	–	–
Venovenous	2.05	1.72–2.44	< 0.001	1.33	1.06–1.67	0.013
Mixed	0.41	0.15–1.11	0.079	0.45	0.16–1.27	0.132
Size of drainage cannula, Fr	1.07	1.03–1.11	0.001	1.01	0.97–1.06	0.549
Size of return cannula, Fr	1.09	1.04–1.13	< 0.001	1.06	1.01–1.13	0.028
Use of unfractionated heparin	0.56	0.47–0.67	< 0.001	0.64	0.52–0.78	< 0.001
Metabolic acidosis	1.42	1.17–1.72	< 0.001	1.17	0.95–1.43	0.147
Access pressure, mmHg	1.001	1.000–1.002	0.027	1.000	0.998–1.002	0.957
Filter pressure, mmHg	0.995	0.994–0.996	< 0.001	1.006	1.002–1.010	0.007
Effluent pressure, mmHg	0.995	0.994–0.996	< 0.001	1.000	0.998–1.002	0.956
Return pressure, mmHg	0.994	0.993–0.995	< 0.001	0.994	0.990–0.998	0.005

*CI* confidence interval, *ECMO* extracorporeal membrane oxygenation; *HR* hazard ratio

## Discussion

In the present study, we investigated the efficacy and safety of using additional pressure control lines to control the pressure on CRRT by connecting the CRRT device to the ECMO circuit. Our findings suggest that the high positive or negative pressure values of the CRRT were attenuated and the lifespan of the CRRT circuit was significantly increased after using the additional lines on both the inlet and outlet lines of the CRRT device connected into the ECMO circuit. Furthermore, the use of multiple lines was not associated with an increase in hemolysis-related complications.

Patients receiving ECMO often suffer acute kidney injury for a number of reasons, such as severe cardiopulmonary insufficiency requiring ECMO support, use of vasoactive drugs or mechanical ventilation before ECMO, and ECMO-associated systemic inflammation [5]. Although CRRT is a common practice in patients receiving ECMO, determining the vascular access to connect the CRRT device, another extracorporeal circuit, is still a challenging problem. One of three possible connection methods is used to perform CRRT in patients on ECMO [7]. One method is to use a vascular access independent from the ECMO circuit as in patients not receiving ECMO. It is possible to control the ultrafiltration via CRRT independent of the ECMO hemodynamics. However, there is an inherent risk of complications associated with catheter insertion; the use of an anticoagulant during ECMO in particular increases the risk of bleeding complications [14]. Another method is to connect the hemodiafilter into the ECMO circuit without an in-line CRRT device and control the ultrafiltration using an intravenous infusion pump. Although this is relatively simple and can avoid complications associated with an additional catheter insertion, several studies reported errors in controlling ultrafiltration via an infusion pump. In addition, since the pressure of the hemodiafilter circuit cannot be monitored, there is a limit to the early detection of mechanical complications of the circuit [6, 9, 15].

Therefore, the introduction of a CRRT device into the ECMO circuit is widely used in many centers [16]. This method enables precise control of ultrafiltration through the CRRT device and monitoring of pressure parameters on the CRRT circuit. However, one of the major disadvantages of incorporating the CRRT device into the ECMO circuit is the interference of blood flow in the CRRT and ECMO circuits [6]. Blood flow within the ECMO circuit causes excessive positive or negative pressure above the physiological range to the CRRT device, which eventually leads to an interruption of CRRT [6, 8, 9]. There are various suggestions for improvement, such as changing the connection site of the ECMO circuit, adjusting the alarm value of the CRRT software, and using a clamp to control the pressures [8–11], but all are associated with potentially unpredictable consequences [10].

Drawing upon the physical law that reducing the lumen of the conduit increases the resistance and reduces blood flow and pressure, we applied an additional line in which the lumen was narrower than those of the inlet and outlet lines of the CRRT circuit between the CRRT device and the ECMO circuit to modify the excessive pressures. This is supported by Suga et al. who investigated the method of connecting an additional pressure-resistant tube (diameter 1.5 mm) to control the pressure transmitted from the ECMO circuit in an in vitro study [11]. Although the inlet and outlet pressures remained within the safety range for all conditions of ECMO and CRRT flow in the in vitro study, the method was validated in only two clinical cases [11]. In our study with a large number of clinical cases, we found that the excessive positive or negative pressure above the physiological range transmitted from the ECMO circuit to the CRRT circuit could be controlled to remain within the safety range using additional lines on both the inlet and outlet lines of the CRRT device connected into the ECMO circuit without complications. In addition, the filter pressure was confirmed to be within the safety range when connected to the ECMO circuit. Owing to the stability of the CRRT system, a longer lifespan of the CRRT circuit was achieved without complications, compared to those reported in previous studies [10, 17, 18].

The different options for connecting a CRRT device into the ECMO circuit have been described in the literature [6]. Inlet of the CRRT device can be connected to the ECMO circuit before or after the oxygenator or centrifugal pump. Similarly, outlet of the CRRT device can be connected to ECMO circuit before or after the oxygenator or centrifugal pump. In our institution, the outlet line is connected into the negative pressure part of the ECMO circuit based on the method suggested by Rubin et al. [8], although there is a hypothetical risk of air entrainment and recirculation. However, any air entrained in the ECMO circuit would be trapped by the oxygenator and recirculation would likely be negligible compared with the ECMO flow [11]. Similar to our model, Santiago et al. [9] suggested that connecting the inlet and outlet lines of the CRRT device after the centrifugal pump would be safe and effective. However, the connection of the outlet line into the positive pressure part of the ECMO circuit can trigger excessive pressure on the outlet line, generating alarms inside the CRRT machine. Our results indicate that this could be managed by additional tubing on the connection.

This study provides information on the use of additional tubing in connecting a CRRT device to an ECMO circuit, but there are several limitations that should be acknowledged. First, given the retrospective and observational nature of our study, there is a potential risk of selection bias and confounding variables. Treatment group size was unbalanced and allocation was non-randomized, resulting in differences in baseline characteristics between groups. And laboratory tests such as plasma hemoglobin were obtained in only a few patients. However, the data were prospectively collected from all patients receiving CRRT during ECMO support. Second, our study demonstrated the effects of using additional tubing on the pressure values and circuit lifetime of a CRRT device. It is difficult to explain the effect on the ECMO circuit because the pressures on the ECMO circuit were not measure. Third, about three-quarters of the patients included in our study had circulatory failure that required venoarterial extracorporeal life support, and this hemodynamic status influenced physicians to describe a relatively low CRRT blood flow rates. Along with this, anticoagulation strategy was different between group without and with additional pressure control lines. These factors could affect the overall circuit lifespan. However, we think that the absolute blood flow rates and differences of anticoagulation strategy had little effect on comparison of CRRT circuit lifespan depending on use of additional pressure line because the blood flow rates and anticoagulation were similar between group with additional pressure control line at inlet line only and group with additional pressure control line at both inlet and outlet lines, a major interest of our study. Last, we did not compare our connection method with additional tubing to other configurations. A standardized connection method has not been established, and our interest was in using additional tubing to effectively control pressures of the CRRT device. Further studies should evaluate the effects of additional tubing with various connections of the CRRT device into the ECMO circuit.

## Conclusions

The present study suggests that the use of additional tubing can be considered a simple and safe method for pressure control and improvement of filter survival when connecting a CRRT device into an ECMO circuit in adult patients. However, further prospective studies should be conducted to investigate the configuration of the two extracorporeal systems and the effect on ECMO hemodynamics.

## Abbreviations

AKI: Acute kidney injury
APACHE: Acute physiology and chronic health evaluation
CRRT: Continuous renal replacement therapy
CVVHDF: Continuous veno-venous hemodiafiltration
ECMO: Extracorporeal membrane oxygenation
ICU: Intensive care unit
IQR: Interquartile range
